# Retrotransposed gene copies persist under relaxed selection in wild *Spodoptera frugiperda*

**DOI:** 10.1093/molbev/msag124

**Published:** 2026-05-27

**Authors:** Kiwoong Nam, Sylvie Gimenez, Hyerin An, Karine Durand, Melanie Gasser, Sudeeptha Yainna, Fabrice Legeai, Julien Beuzelin, David G Heckel, Sabine Hänniger, Emmanuelle d'Alençon

**Affiliations:** DGIMI, INRAE, Univ Montpellier, Montpellier, France; DGIMI, INRAE, Univ Montpellier, Montpellier, France; DGIMI, INRAE, Univ Montpellier, Montpellier, France; DGIMI, INRAE, Univ Montpellier, Montpellier, France; DGIMI, INRAE, Univ Montpellier, Montpellier, France; DGIMI, INRAE, Univ Montpellier, Montpellier, France; INRAE, UMR-IGEPP, Bioinformatics Platform for Agroecosystems Arthropods, Campus Beaulieu, Rennes 35042, France; Everglades Research & Education Center, University of Florida Institute of Food and Agricultural Sciences, Belle Glade, FL 33430, USA; Entomology Department, Max Planck Institute for Chemical Ecology, Jena, Germany; Entomology Department, Max Planck Institute for Chemical Ecology, Jena, Germany; Institute for Biodiversity, Ecology and Evolution, Friedrich Schiller University Jena, Jena, Germany; DGIMI, INRAE, Univ Montpellier, Montpellier, France

**Keywords:** copy number variation, individually assembled genome, intact ORF, long interspersed nuclear element, retrotransposition, *Spodoptera frugiperda*

## Abstract

Copy number variations (CNVs) are major sources of genetic variation, affecting large portions of genomes. Typical CNVs are known to have short evolutionary half-lives due to deleterious effects, with documented events of adaptive evolution. However, it is not known whether the same trends apply to CNVs preserving intact Open Reading Frames (iORF-CNVs), which can encode functional proteins. Here, we investigate the mechanistic origins and evolutionary dynamics of iORF-CNVs in *Spodoptera frugiperda*, a globally distributed pest species where CNV-mediated adaptive evolution has been reported. Using PacBio HiFi reads, we generated high-quality, individually assembled genomes for 36 field-collected individuals and identified 349 genes with polymorphic iORF-CNVs. These elements are short, intronless, and display molecular signatures of LINE-mediated retrotransposition, including *cis-* and *trans-*acting effects. A majority of iORF-CNVs (68.0% to 77.28%) exhibited at least one molecular signature of retrotransposition. iORF-CNVs showed lower divergence at the first and second codon positions than at the third codon positions, but they had higher nonsynonymous-to-synonymous polymorphism ratios than non-iORF-CNV genes, implying purifying selection with relaxed constraint. Orthology analyses indicated that these CNVs originate from genes under weak evolutionary constraint, while transcriptomic and promoter motif analyses revealed that many are transcribed and retain regulatory features. We conclude that observed iORF-CNVs are generated through retrotransposition from weakly constrained genes and that the duplicates are selectively maintained for coding potential, albeit under reduced purifying selection compared with non-iORF-CNV genes. These results imply that pseudogenization is not an inevitable evolutionary fate of retrotransposed gene copies when the open reading frame is preserved.

## Introduction

Copy number variations (CNVs) are major sources of genetic variation. In humans, for example, CNVs affect nearly 30% of the genome (Zhang et al. 2009), while single nucleotide variations (SNVs) comprise 84.7 Mb (Auton et al. 2015), corresponding to only 2.73% of the 3.1 Gb human genome. CNVs have been proposed as substrates for adaptive evolution through the beneficial effects of changes in gene expression resulting from different copy numbers (Spealman et al. 2025). Duplicated genes can contribute to evolutionary innovations by relaxing the evolutionary constraints on the original copies (Ohno 1970; Hughes 2002; Innan and Kondrashov 2010) or by generating diverse substrates that can be selectively targeted (Hoile et al. 2025), for example, for the immune system (Maizels 2005) or for detoxification (Calla et al. 2017). The deleterious effects of CNVs have also been extensively documented, particularly in the context of human disease (Girirajan et al. 2011; Pös et al. 2021).

The evolutionary forces acting on the individual duplicated gene copies present within CNVs have been extensively studied over the past two decades, revealing a general trend. Population genetic analyses of polymorphic CNVs suggest that duplicated copies typically have much shorter evolutionary half-lives than SNVs, likely due to the deleterious effects (Redon et al. 2006; Emerson et al. 2008; Sudmant et al. 2010; Schrider et al. 2011, 2013; Zarrei et al. 2015; Zhang et al. 2021; Zhang and Tautz 2022; Fang and Edwards 2024), while effectively neutral CNVs may also constitute a fraction of the standing variation. There are also well-documented instances of adaptive evolution involving CNVs (Lye and Purugganan 2019; Sirén et al. 2021; Li et al. 2023; Jayakodi et al. 2024), but, given that the majority of CNVs involve truncated genes, it is perhaps unsurprising that most CNVs exhibit non-adaptive consequences.

**Figure 1:**
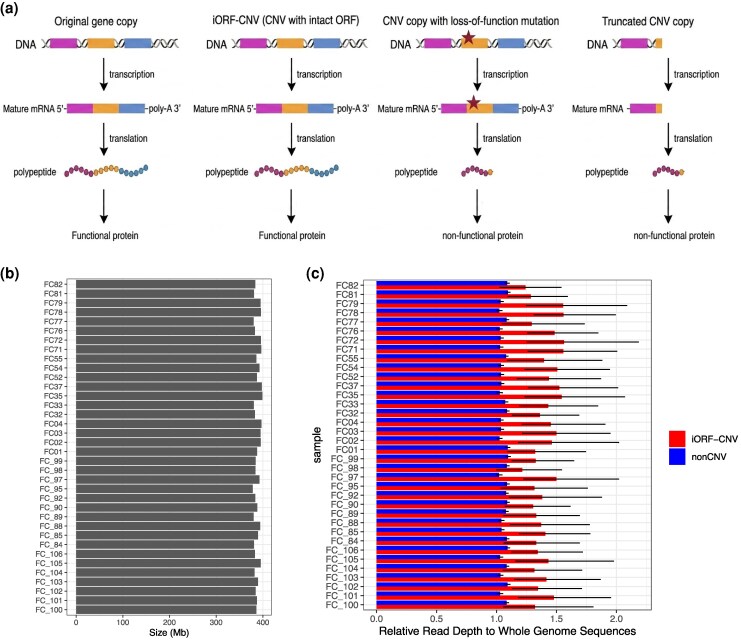
a) Schematic representation of iORF-CNVs. If a duplicated gene copy maintains an intact open reading frame (iORF-CNV), a functional protein can be generated. Conversely, nonfunctional proteins are produced if the duplicated copy contains a loss-of-function mutation (indicated by a red star), such as a nonsense or frameshift mutation, or if the duplicated copy is truncated. b) The size of individually assembled genomes. c) The read depth of iORF-CNVs and non-iORF-CNV genes normalized by the genomic read depth.

However, it is not clear if generalization can be extended to the CNV copies with intact Open Reading Frame (iORF-CNV), which can encode functional protein-coding sequences (Fig. 1a). iORF-CNVs caused by retrotransposition have been reported in certain species (Gao et al. 2019; Nguyen et al. 2024). If retrotransposition is a general mechanism of iORF-CNVs and iORF-CNVs generally lack promoter sequences during retrotransposition, iORF-CNVs would not be functionally constrained and would eventually become pseudogenes. Alternatively, if iORF-CNVs are transcribed but exert deleterious effects by altering gene expression levels, pseudogenization might also be favored through positive selection of nonsense or frameshift mutations. Conversely, if changes in protein sequences are more deleterious than changes in gene expression, iORF-CNVs may be subject to functional constraint. Importantly, we cannot exclude the possibility that the observed iORF-CNVs are generally targets of positive selection.

Retro-CNVs are believed to be caused by transposable elements (TEs) through retrotransposition of cytoplasmic processed transcripts. In particular, Long Interspersed Nuclear Elements (LINEs) exert *trans*-effects of retrotransposition when the reverse transcriptase recognizes the poly-A tails of cytoplasmic transcripts (Doucet et al. 2015), leading to the retro-insertion of these transcripts into the genome (Esnault et al. 2000). Such retrotransposed copies exhibit distinct footprints, including being intronless and having poly-A tails with target site duplications at both ends of the inserted gene. LINEs also exhibit *cis*-effects, where genes located near LINEs are co-transcribed in a single transcript and co-inserted into the genome (Moran et al. 1999). This LINE-mediated transduction has been frequently observed (Goodier et al. 2000; Kim et al. 2019; Schloissnig et al. 2025). Theoretically, iORF-CNVs can be generated through retrotransposition either by the *trans-* or *cis*-effects of LINEs. Alternatively, iORF-CNVs may also arise through non-retrotransposition processes, such as non-allelic homologous recombination, non-homologous end joining during DNA repair, or errors occurring during DNA replication (Hastings et al. 2009), particularly if most CNVs are produced by non-allelic homologous recombination (Schrider and Hahn 2010).

To address the formation mechanisms and evolution of iORF-CNVss and the evolution of iORF-CNVs, we focus on the fall armyworm (*Spodoptera frugiperda*; Lepidoptera; Insecta), a highly polyphagous pest insect species that feeds on more than 353 plant species across 76 families (Montezano et al. 2018). It comprises two host-associated strains, the corn strain and the rice strain, named after their preferred host plants in their native range across the Americas (Pashley 1986). Since its initial report in West Africa in 2016 (Goergen et al. 2016), invasive *S. frugiperda* has rapidly expanded its range across Africa, Asia, Oceania, and, most recently, Europe. Invasive *S. frugiperda* was identified as belonging to the corn strain (Yainna et al. 2022; Durand et al. 2024), thereby causing severe losses in maize production, for example, reducing yields by between 21% and 53% in sub-Saharan Africa (Day et al. 2017). Hence, the spread of *S. frugiperda* is exerting significant pressure on global agriculture (Overton et al. 2021).

In *S. frugiperda*, CNVs are overrepresented in genes involved in interactions with host plants, particularly detoxification and digestion genes (Gouin et al. 2017). Compared to monophagous lepidopteran species, *S. frugiperda* harbors an increased copy number of detoxification genes followed by diversification (Gouin et al. 2017). This diversification may have contributed to the ability to metabolize a broad range of plant defense compounds from diverse plant species. Additionally, positive selection for increased copy numbers of insecticide resistance genes has been reported in populations from Puerto Rico (Gimenez et al. 2020), where insecticides are intensively used for maize seed production (Belay et al. 2012).

In this study, we investigated the mechanisms and evolutionary patterns of iORF-CNV polymorphisms in *S. frugiperda* using high-quality, individually assembled genomes from a natural population. These assemblies provide a maximally complete representation of each organism's genetic makeup, capturing structural variations, including CNVs. Moreover, these assemblies enable detailed sequence analyses for each individual, allowing the identification of polymorphic patterns that are potentially linked to the mechanistic origins and evolutionary forces underlying iORF-CNVs.

Our research was structured into three primary objectives. First, we generated individual genome assemblies from 36 samples to create a dataset that robustly represents the population, followed by the identification of genes showing iORF-CNVs. Second, we investigated the molecular mechanisms responsible for the generation of these iORF-CNVs. Third, we investigated the evolutionary fate of the identified iORF-CNVs.

## Results

### Sequencing and genome assembling

We first established a bioinformatics pipeline to generate high-quality individual genome assemblies using one-sixth of a PacBio HiFi flow cell, optimizing the approach with a laboratory colony before applying it to a large number of samples from a natural population. High-molecular-weight gDNA was extracted from a single insect of a laboratory *S. frugiperda* strain (Gouin et al. 2017) and PacBio HiFi sequencing was performed, yielding 28 Gb from a single flow cell. The one-sixth subset of the flow cell was 4.6 Gb, which corresponds to 11.5X coverage for the 400 Mb genome (Gouin et al. 2017; Durand and Nam 2025). Among three assemblers, including wtdbg2 (Ruan and Li 2020), raven (Vaser and Šikić 2021), and hifiasm (Cheng et al. 2021), wtdbg2 produced the highest BUSCO scores and contiguity (Table S1). Consequently, we selected wtdbg2 as the assembler. When the resulting assembly was polished three times with racon (Vaser et al. 2017), the number of “Single and Complete” BUSCOs increased from 5,134 to 5,148, a value even higher than that of the ver7 reference genome assembly (5,103) (Fiteni et al. 2022). Therefore, we established a bioinformatics pipeline consisting of wtdbg2 followed by three rounds of racon polishing for genome assembly.

Subsequently, high-molecular-weight gDNA was extracted from 52 fresh insect samples collected in a maize field in Florida (see “Material and methods” for details). In total, 36 samples yielded >4 µg of non-degraded gDNA (Figure S1). PacBio HiFi sequencing was performed on these 36 samples using six flow cells, with six samples multiplexed per flow cell. The average sequencing throughput per sample was 4.61 Gbp (11.53X coverage, assuming a genome size of 400 mb), with a range from 3.12 Gbp (7.8X) to 10.39 Gbp (25.98X) (Figure S2). The size of the individually assembled genome sequences ranged from 378.1 to 398.8 Mb (Fig. 1b), with N50 values ranging from 352 Kb to 3.710 Mb, and an average of 1.168 Mb (Figure S3). These assembly sizes closely matched the genome size measured by flow cytometry, which was 396 ± 3 Mb (Gouin et al. 2017). Assembly sizes exhibited a bimodal distribution according to sex (ZZ in males and ZW in females in Lepidoptera), suggesting that the presence of the W chromosome increased the assembly size in females (Figure S4).

The proportion of Complete and Single-copy BUSCO genes was consistently higher than 95% in all samples (95.8% to 97.6%), except for one sample (FC72, 93.9%), which was comparable to the reference genome assembly (96.5%) (Figure S5). The proportion of Complete and Duplicated BUSCO genes ranged between 0.4% and 1.4%, which was also comparable to the reference genome assembly (1.0%) (Figure S5). Assuming that highly similar sequences within a single assembly can result from artificial duplications, we searched for sequence pairs longer than 100 kb with over 98% similarity through self-alignment. No assembly exhibited such cases, indicating that there were no obvious artificial duplications. Furthermore, the mappings of each assembly to the reference genome assembly show clear collinearity (Figure S6).

We annotated genes for each genome assembly by mapping 14,679 unique protein sequences from the RefSeq dataset, which was originally derived from the repeat-masked reference genome. The proportion of mapped genes ranged from 99.30% to 99.54% of the total RefSeq genes among the 36 assemblies. We filtered out those with protein sequence identities below 95% or coverage below 95% and discarded mappings containing internal stop codons to ensure the identification of genes with intact ORFs. After this filtering, the remaining genes ranged from 7,973 to 10,227, corresponding to 54.11% to 68.68% of the total RefSeq genes (Figure S7). Since the varying number of genes might affect downstream analyses, we analyzed only the genes identified in at least two-thirds of the total samples (ie 24 samples), resulting in 7,945 genes.

### Identification of iORF-CNVs

To identify polymorphic iORF-CNVs, we analyzed variation in the number of mappings among genome assemblies. Since misassemblies could affect the number of mappings, we first analyzed the mapping of lepidopteran BUSCO genes, assuming that each should map only once per genome. Although BUSCO genes are not necessarily absolute single-copy genes (Simão et al. 2015), multiple mappings were assumed to be potential indicators of misassembly. The vast majority of BUSCO genes (98.85% of 5,224 genes) had duplications from fewer than four samples (Figure S8). Thus, multiple mappings observed in at least four independent genome assemblies were considered unlikely to result from misassemblies.

Multiple mappings from at least four samples were observed in 371 RefSeq genes. To exclude TEs from candidate iORF-CNVs, we removed genes where 90% of the sequence was TE-derived and genes with TE-like annotations. After this filtering, 349 RefSeq genes were retained, and we considered that these genes exhibited iORF-CNVs in the population (see Table S2 for the full list). These 349 genes were clustered into 288 distinct groups based on the same sequence identity and coverage criteria used for mapping (95% sequence identity and 95% coverage).

To further validate the presence of polymorphic iORF-CNVs, we calculated the read depth of the mappings against the reference genome. The polymorphic iORF-CNVs consistently exhibited higher read depth than the other genes by 11.8% to 52.3% (FDR corrected *P-*values < 0.005, permutation test with 1,000 replications), supporting the increased copy numbers of the identified iORF-CNVs (Fig. 1c). The RefSeq genes showing iORF-CNVs spanned a total of 2,647,800 bp, accounting for 0.642% of the reference genome (Figure S9). This length is substantially lower than the total length of SNVs identified from the HiFi reads (24,714,105 bp), implying that the genomic proportion occupied by iORF-CNVs is smaller than that affected by SNVs.

The iORF-CNVs included 12 genes encoding cuticle proteins, 12 chorion genes, and 5 trypsin genes (Table S3), along with 196 genes of unknown function. In total, 17 Gene Ontology terms were overrepresented in the list of polymorphic iORF-CNVs (Table S4), including three chorion-related ones, such as “chorion-containing eggshell formation” (GO:0007304, odd ratio = 322.49, FDR-adjusted *P-*value = 3.32 × 10^−14^). A cuticle-related Gene Ontology term, “structural constituent of cuticle” (GO: 0042302, odds ratio = 4.22, FDR-adjusted *P-*value = 3.02 × 10^−3^), was also overrepresented. This result indicates a nonrandom association between the iORF-CNVs and their functions.

### Retrotransposition as a cause of iORF-CNVs

**Figure 2:**
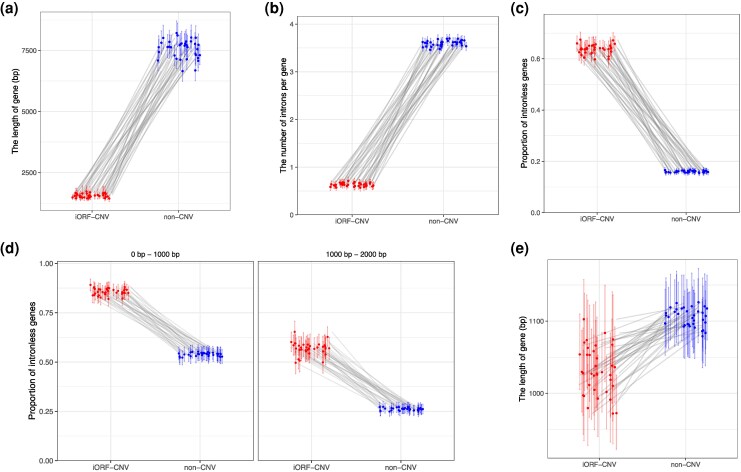
The structure of iORF-CNV. Comparison of a) gene length, b) number of introns, c) proportion of intronless genes, d) proportion of intronless genes within specific length bins, and e) gene length of single-exon genes between genes with multicopy iORF-CNVs (red) and non-iORF-CNV genes (blue) in a genome for each sample. Error bars represent 95% confidence intervals calculated from bootstrapping based on 1,000 resamplings of 100 kb genomic windows.

The average length of the iORF-CNVs in each assembly ranged from 1,352 to 1,637 bp, substantially shorter than that of non-iORF-CNVs, which ranged from 6,654 to 8,212 bp, by a factor of 4.43 to 5.65 (FDR-adjusted *P-*values < 2.2 × 10^−16^ for each genome assembly, two-tailed Wilcoxon signed-rank test) (Fig. 2). Similarly, iORF-CNVs had fewer introns (range: 0.515 to 0.708) than non-iORF-CNV genes (range: 3.461 to 3.698), by a factor of 5.03 to 7.05 (FDR-adjusted *P-*values < 2.2 × 10^−16^). The proportion of intronless ORF-CNVs (50.79% to 67.46%) was significantly higher than that of non-iORF-CNV genes (15.45% to 16.78%, FDR-adjusted *P-*values < 2.2 × 10^−16^, two-tailed Fisher's exact test). To control for the effect of gene length, we analyzed the proportion of intronless genes within specific size bins. In the 0 to 1,000 bp range, the proportion of intronless ORF-CNVs (81.98% to 89.12%) remained significantly higher than that of non-iORF-CNV genes (49.66% to 65.32%) (FDR-adjusted *P-*values < 2.2 × 10^−16^). Similarly, within the 1,000 to 2,000 bp range, the intronless proportion of ORF-CNVs (52.14% to 62.88%) was also significantly greater than that of non-iORF-CNV genes (26.69% to 31.49%) (FDR-adjusted *P-*values < 1.99 × 10^−13^). Among the intronless genes, iORF-CNVs were consistently shorter than non-iORF-CNV genes across all samples with statistically significant differences (FDR-adjusted *P-*value*s* < 0.1), except for one sample (FDR-adjusted *P-*values =0.1907). However, the magnitude of these differences was relatively small, ranging only between 0.23% and 17.18%. This result suggests that the observed difference in gene length is primarily driven by intron numbers, proposing the possibility that a substantial proportion of iORF-CNVs were generated through retrotransposition.

To investigate the genomic context of the identified polymorphic iORF-CNVs, we mapped a pair of 100 kb flanking sequences of each iORF-CNV gene from the individual assemblies back to the reference genome. These flanking sequences of all 349 iORF-CNV genes were successfully localized to specific genomic coordinates. In the vast majority of cases (91.98%; 321/349), the flanking sequences mapped to adjacent regions in the reference genome without intervening genes. This result suggests that these iORF-CNVs originated from insertion events into non-genic regions in the reference individual, although the possibility of gene excision in the reference genome cannot be entirely excluded. Among these insertions, 63.6% (222/349) shared the same flanking sites across multiple individuals, indicating that they represent shared alleles derived from common ancestral insertion events. The singleton insertions (*n* = 99) are noticeable, as these insertions indicate that single genes are inserted into different genomic sites multiple times independently.

**Figure 3:**
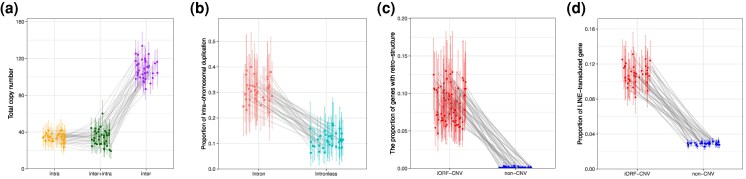
The possibility of retrotransposition causing polymorphic iORF-CNVs. a) Number of iORF-CNV copies classified as exclusively intrachromosomal, exclusively interchromosomal, or a combination of inter- and intrachromosomal duplications for each sample. b) Proportion of exclusively intrachromosomal duplications among intronless and intron-containing iORF-CNVs. c) Proportion of iORF-CNVs exhibiting retrotransposition-derived structural features, including the presence of target site duplications, the intact gene body, and a poly-A tail. d) Proportion of iORF-CNVs associated with LINE-mediated transduction events. Error bars represent 95% confidence intervals calculated from bootstrapping based on 1,000 resamplings of 100 kb genomic windows.

When the genomic location of each iORF-CNV copy was examined, 75.86% to 84.41% of the polymorphic iORF-CNV copies involved interchromosomal duplications (Fig. 3a). Among them, intronless copies had lower proportions of exclusive intrachromosomal duplications (6.33% to 17.91%) than intronic copies (22.22% to 40%), with statistical significance observed in 34 out of 36 assemblies (FDR-adjusted *P-*values < 0.1, permutation test with 1,000 replications) (Fig. 3b). This result indicates that the iORF-CNVs frequently involve interchromosomal duplications with a stronger tendency for intronless genes, consistent with the expectation that a substantial proportion of the iORF-CNVs originated through retrotransposition.

Then, we tested the *trans*-effects of LINEs, which typically result in retrotransposed sequences flanked by poly-A tails and target site duplications (Richardson et al. 2014). To test whether the polymorphic iORF-CNVs exhibit these structures, we searched for the presence of poly-A tails and target site duplications at both ends of each iORF-CNV or non-iORF-CNV gene for each genome assembly. To ensure stringency, we considered only poly-A tails that were ≥20 bp in length and target site duplications that were ≥5 bp. iORF-CNVs showed a higher proportion of these structures (4.71% to 8.25%) than non-iORF-CNV genes (0.195% to 0.273%) (FDR-corrected *P-*value < 2.277 × 10^−18^, two-tailed Fisher's exact test) (Fig. 3c). This result supports the hypothesis that a substantial proportion of the iORF-CNVs originated through retrotransposition via *trans*-effects of LINE.

We also tested LINE-mediated transduction through *cis-*effects. iORF-CNV genes exhibited higher proportions of cases in which the gene is flanked by a LINE sequence (8.20% to 13.12%) than non-iORF-CNV genes (2.55% to 3.21%) by a factor of 2.77 to 4.74 (FDR-adjusted *P-*values < 1.7 × 10^−11^, Fig. 3d). This result supports the role of the *cis-*effect of LINE through transduction.

To quantitatively estimate the extent to which retrotransposition contributes to the genesis of iORF-CNVs, we calculated the proportion of iORF-CNVs satisfying three distinct retrotransposition hallmarks across all 36 genome assemblies, including intronless, interchromosomal duplication, and LINE-mediated insertion footprints. A substantial majority of iORF-CNVs, ranging from 68.0% to 77.28%, exhibited at least one of these indicators (Figure S10). Notably, a significant proportion (35.68% to 43.44%) satisfied at least two criteria, while 8.75% to 12.80% met all three conditions concurrently. These results suggest that retrotransposition is one of the major drivers of iORF-CNV formation.

### Shared evolutionary forces determining iORF-CNV and TE element distribution

**Figure 4:**
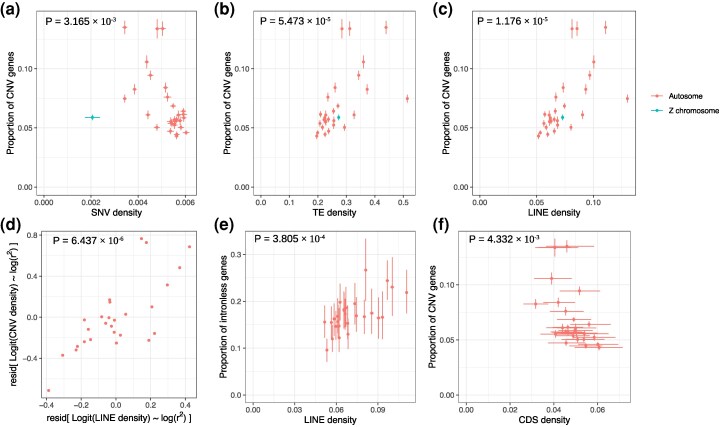
The distribution of iORF-CNVs and transposable elements. Correlation of iORF-CNV density calculated for each chromosome with a) SNV density, b) transposable element density, and c) LINE density. d) Partial correlation analysis between LINE density and iORF-CNV density, controlling for the effect of linkage disequilibrium (estimated by *r^2^*). e) Correlation between LINE density and the proportion of intronless genes. f) Correlation between protein-coding sequence density and iORF-CNV density. Error bars represent standard errors, calculated from sample-level statistics across genomes.

Then, we inferred the evolutionary forces determining the spatial distribution of iORF-CNVs along the genome. The iORF-CNV density calculated for each chromosome was negatively correlated with SNV density (Spearman's ρ = −0.535, *P-*value = 0.003165) (Fig. 4a), suggesting that the distribution of CNVs is shaped by evolutionary forces different from those affecting point mutations. In contrast, iORF-CNV density exhibited a positive correlation with both total TE (*ρ* = 0.30, *P-*value = 5.473 × 10^−5^) and LINE densities (ρ = 0.732, *P-*value = 1.176 × 10^−5^) (Fig. 4b and c). This result suggests that the distributions of TEs and polymorphic iORF-CNVs can be influenced by the same evolutionary forces, such as shared insertional preferences.

We indirectly tested this possibility using an autosomal correlation test between iORF-CNV and LINE densities while controlling for the effect of purifying selection. We assumed that linkage disequilibrium can be used as a proxy for the inefficacy of selection, as linked selection reduces local effective population size (Smith and Haigh 1974; Charlesworth 1996). The SNV density showed a negative correlation with the *r*^2^ of allele frequencies, used as a measure of linkage disequilibrium strength (ρ = −0.5360, *P-*value = 0.003133), suggesting that this assumption is valid. iORF-CNV density was positively correlated with *r*^2^ (*ρ* = 0.5517, *P-*value = 0.00225). After accounting for the effects of *r*^2^ on both iORF-CNV density and LINE density using partial regression, the positive correlation between LINE density and iORF-CNV density remained (*P-*value = 6.437 × 10^−6^, Fig. 4d), supporting the possibility of a shared insertional preference between LINE and iORF-CNVs.

Shared insertional preferences between LINEs and iORF-CNVs were further tested with a focus on intronless genes. We assumed that most intronless genes experienced retrotransposition over long evolutionary periods (Grzybowska 2012), during which detectable LINE insertions occurred in the genome. The LINE density was positively correlated with the proportion of intronless genes in the reference genome (*ρ* = 0.6453, *P-*value = 0.0003805, Fig. 4e), supporting this possibility. Coding sequence density was negatively correlated with iORF-CNV density (*ρ* = −0.6404, *P-*value = 0.0004332, Fig. 4f), suggesting that regions with lower coding density offer higher opportunities for both LINE and iORF-CNV insertion than regions with lower density.

### Selective constraints on iORF-CNVs

**Figure 5:**
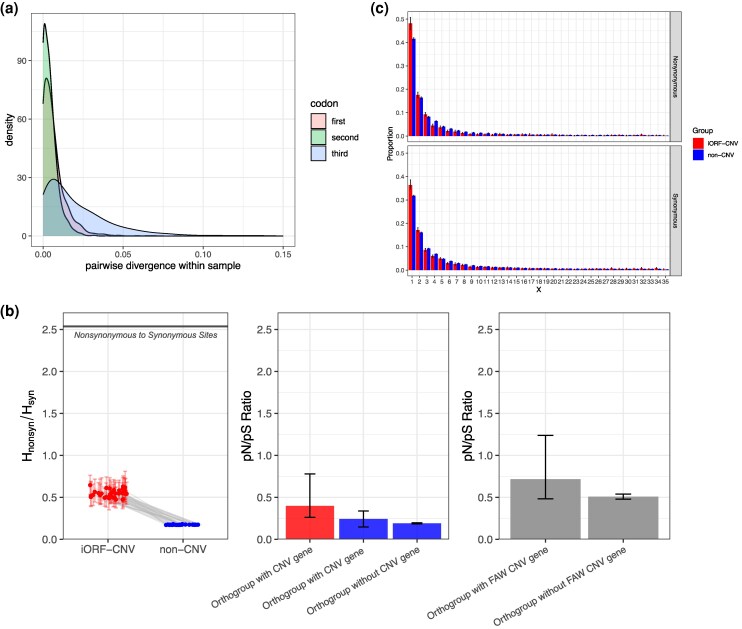
Evolutionary forces underlying polymorphic iORF-CNVs. a) Density plot of pairwise nucleotide divergence among iORF-CNV copies within a single genome, calculated separately for the first, second, and third codon positions. b) (Left) Ratio of nonsynonymous missense to synonymous heterozygous positions for each sample in iORF-CNV genes versus non-iORF-CNV genes. Horizontal lines indicate the ratio of nonsynonymous missense to synonymous sites in the same gene sets. (Middle) Ratio of nonsynonymous missense to synonymous polymorphisms in iORF-CNV genes, non-iORF-CNV genes within orthogroups containing iORF-CNVs, and genes from orthogroups without iORF-CNVs. (Right) Ratio of nonsynonymous missense to synonymous polymorphisms in *S. exigua*, comparing orthologous genes with or without iORF-CNVs in *S. frugiperda*. c) Folded site frequency spectra of nonsynonymous and synonymous polymorphisms in iORF-CNV and non-iORF-CNV genes. Error bars represent 95% confidence intervals calculated from bootstrapping based on resamplings of genes.

To test the evolutionary constraints on the iORF-CNVs, we calculated the pairwise divergence of coding sequences among iORF-CNV copies originating from the same genes within a single assembly. Third codon positions exhibited higher divergences (2.371%, 95% CI: 2.271% to 2.471%) than first (0.686%, 95% CI: 0.665% to 0.706%) or second codon positions (0.429%, 95% CI: 0.418% to 0.441%) (Fig. 5a), which is expected for evolutionarily conserved coding genes. iORF-CNVs displayed a higher pN/pS (nonsynonymous to synonymous polymorphisms ratio) (0.4722 to 0.6454) than non-iORF-CNV genes (0.1708 to 0.1813, FDR-corrected *P-*value < 2.2 × 10^−16^; Fisher's exact test) (Fig. 5b). However, these pN/pS values are much lower than the ratio of nonsynonymous to synonymous sites (2.536, 95% CI: 2.534 to 2.539), implying underrepresentation of nonsynonymous variations at iORF-CNVs. These results suggest that polymorphic iORF-CNVs are under selective constraints, but to a lesser extent than non-iORF-CNV genes.

The lower pN/pS observed in iORF-CNVs than in non-iORF-CNV genes may result from relaxed selective constraints following duplication. Alternatively, and not mutually exclusively, this pattern could indicate that the iORF-CNVs originated from genes that already had lower selective constraints before duplication than the rest of the genome. To test these possibilities, pN/pS was calculated from the iORF-CNVs and non-iORF-CNVs genes within the same orthogroups. iORF-CNVs exhibited a higher pN/pS ratio (0.3983; 95% CI: 0.2616 to 0.7797) than non-iORF-CNV genes within the same orthogroups (0.2418; 95% CI: 0.1474 to 0.3375; *P-*value < 2.2 × 10^−16^; odds ratio = 1.647) (Fig. 5c). These non-iORF-CNV genes, in turn, had higher pN/pS than the genes in orthogroups without iORF-CNVs (0.1904; 95% CI: 0.1847 to 0.1967; *P-*value < 2.2 × 10^−16^; odds ratio = 1.270). This result supports the hypothesis that iORF-CNVs originated from genes already under lower constraints and, also, that the selective constraint was further reduced after duplication.

To test this hypothesis further, we analyzed resequencing data from 16 samples of *S. exigua*. Orthogroups containing *S. frugiperda* iORF-CNV genes exhibited higher pN/pS in *S. exigua* (0.7152, 0.4815 to 1.2381 of 95% CI) than orthogroups without *S. frugiperda* iORF-CNV genes (0.5088, 0.4777 to 0.5391 of 95% CI) (*P-*value = 0.005463, odds ratio = 1.406) (Fig. 5c). This result also support that iORF-CNVs originate from genes already under weak selective constraint prior to duplication.

The increased pN/pS in iORF-CNVs could also be explained if a significant proportion of CNV copies are experiencing positive selection through the increase in the frequency of nonsynonymous alleles at the time of sample collection. If true, we expect iORF-CNVs to have a higher proportion of derived alleles for nonsynonymous polymorphisms than non-iORF-CNV genes. However, we observed no clear difference in the folded site spectrum between iORF-CNVs and non-iORF-CNV genes, except for singleton polymorphisms (Fig. 5d), which should be primarily determined by demographic events (Cubry et al. 2017). This result suggests that positive selection is unlikely to be the main evolutionary force driving the increased pN/pS in iORF-CNVs.

### Promoter sequences of iORF-CNVs

**Figure 6:**
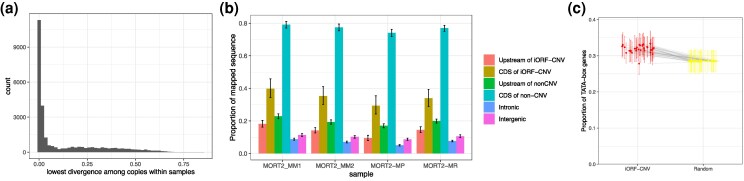
Transcription of iORF-CNVs. a) Distribution of the lowest pairwise divergence among 300 bp upstream sequences of iORF-CNV copies within a genome. b) Proportion of sequences with mapped reads in 300 bp upstream regions of iORF-CNV and non-iORF-CNV genes, as well as intronic, intergenic, and protein-coding sequences. c) Proportion of 300 bp upstream regions containing TATA-box motifs for iORF-CNV (red) and 1,000 randomly selected genomic sequences (yellow) for each sample. Error bars represent 95% confidence intervals calculated from bootstrapping based on resamplings of b) 100 kb windows or c) genes.

Because evolutionary constraint on iORF-CNVs is expected only if they are transcribed, we tested the presence of potential transcriptional regulatory elements. We focused on the 300 bp upstream flanking sequences, which are typically considered the minimum length required to identify promoter regions in eukaryotes (Lai et al. 2019). A total of 60.32% of copies within iORF-CNVs exhibited 300 bp of upstream flanking sequences with <5% sequence divergence from the flanking sequences of other copies within a genome (Fig. 6a), suggesting the preservation of regulatory elements in these upstream regions, which might be involved in transcription.

Next, we tested whether iORF-CNVs and their 300 bp upstream sequences are indeed transcribed by analyzing transcriptome data from four larvae. Coding sequences of non-iORF-CNV genes showed a higher proportion of sequences with mapped RNA-seq reads (74.13% to 79.16%) than iORF-CNVs (29.36% to 39.71%) (Fig. 6b). Both were higher than the proportions observed for intronic (5.03% to 8.75%) and intergenic regions (8.69% to 11.38%), suggesting that a subset of iORF-CNVs are indeed transcribed. The 300 bp upstream sequences also had a higher proportion of sequences with mapped reads than intronic and intergenic sequences for both iORF-CNVs (9.42% to 18.18%) and non-iORF-CNV genes (17.10% to 22.89%) for all investigated samples. This result indicates that iORF-CNVs and their upstream regions are transcribed more abundantly than intronic or intergenic regions.

To assess promoter functionality more directly, we searched for canonical TATA-box motifs (TATAWAW (Donczew and Hahn 2017)) in the 300 bp upstream regions. Non-iORF-CNV genes had a higher proportion of genes with TATA-box motifs than randomly chosen 1,000 positions, by 21.03% to 29.50% (FDR-corrected *P-*value < 2.2 × 10^−16^) (Figure S11). This result suggests that TATA-box motifs can be used as indicators of promoters, even though a significant proportion of promoters are not expected to contain a TATA-box motif. The 300 bp upstream sequences of iORF-CNVs had higher proportions of TATA-box motifs (30.46% to 33.37%) than random sequences (28.17% to 28.65%, Fig. 6c) with statistical support from all samples (FDR-corrected *P-*value*s* < 0.1), with a single exception (27.83%, FDR-corrected *P-*value = 0.627). This result supports that the upstream sequences of iORF-CNVs play a role in promoter activity.

### Fixation of retrotransposed genes

Next, we investigated the evolutionary fate of iORF-CNV copies. Because we have excluded rarely occurring iORF-CNVs to minimize false positives, it was not possible to directly estimate the fixation probabilities. Instead, we focused on intronless genes, assuming that these genes experienced past events of retrotransposition. If a retrotransposed copy becomes fixed and the original is lost or pseudogenized, synteny can be disrupted among genes, particularly given the frequent interchromosomal duplications observed for iORF-CNVs (Fig. 3a and 3b).

To test this possibility, we first identified orthologous chromosomes among *S. frugiperda*, *S. litura*, and *S. exigua* using 5,286 BUSCO genes. Almost no deviations in orthologous chromosomes among these three species were observed from the BUSCO genes (Figure S12), as previously reported (Wright et al. 2024). Given that *S. frugiperda* and *S. litura* are sister taxa with *S. exigua* as an outgroup, we assume that, if all or a subset of *S. frugiperda* genes are intronless while all genes in orthologs of the other species retain introns, this intron loss occurred specifically in the *S. frugiperda* lineage after its divergence from the lineage leading to *S. litura*. These intron-losing genes exhibited 23.4-fold and 29.7-fold greater synteny deviation than intronic and constitutively intronless genes, respectively (Figure S13, with examples), supporting the fixation of duplicated copies to a detectable extent.

## Discussion

While the general mechanisms and evolutionary patterns of CNVs are well documented, the evolutionary dynamics of CNVs preserving intract Open Reading Frames (iORF-CNVs) remain insufficiently understood, leaving a gap in our comprehensive understanding of CNV evolution. To address this issue, we generated 36 individually assembled genomes from field-collected *S. frugiperda* samples using approximately 10X coverage for each sample. The sizes of all resulting assemblies are comparable to the measurements from flow cytometry, consistent with the conserved genome size within the species (Durand and Nam 2025). The BUSCO statistics reveal that these assemblies have comparable quality to the reference genome assembly, despite the relatively low coverage. We identified 349 RefSeq genes exhibiting polymorphic iORF-CNVs. The relatively higher mapping read depth of these iORF-CNVs than non-CNVs implies that the identified iORF-CNVs are robust.

These iORF-CNVs tend to be short, intronless, and display hallmarks of retrotransposition, including signatures of both *cis-* and *trans-*effects of LINEs. A majority of iORF-CNVs (68.0% to 77.28%) exhibited at least one molecular signature of retrotransposition including intronless, interchromosomal duplication, and footprints of LINE-mediated insertion, showing that retrotransposition is one of the major mechanisms of the formation of iORF-CNVs. Ortholog analysis with pN/pS calculations revealed that these iORF-CNVs are evolutionarily constrained, albeit under relaxed purifying selection, and originated from genes already subject to weak selective pressure. Additionally, a substantial number of iORF-CNV copies retained upstream regulatory sequences associated with transcriptional activity, indicating a potential requirement for functional conservation. Synteny analysis further showed that a notable proportion of retrotransposed copies have been fixed in the population. Together, we conclude that a substantial proportion of iORF-CNVs originates from genes under weaker purifying selection, through retrotransposition with both *cis-* and *trans-*effects of LINEs, while still being evolutionarily constrained to a lesser extent.

The evolutionary constraint observed on iORF-CNVs suggests that changes in amino acid sequences caused by mutations may be more deleterious than potential alterations in gene expression due to copy number variation. The pairwise divergence among iORF-CNV copies within a genome was 2.371% (2.271% to 2.471% of 95% CI) at third codon positions, indicating that the polymorphic copy numbers have existed long enough to generate this divergence, although the divergence estimation can be influenced by heterozygous nucleotides. Assuming a mutation rate of 2.9 × 10^−9^ per site per generation (Keightley et al. 2015), the average age of the iORF-CNV copies would be approximately 4 million generations (2.371%/(2.9 × 10^−9^ × 2)), suggesting that the heterogeneous copy numbers of iORF-CNV genes have been tolerated over this period. We postulate the possibilities that fitness effects have not been severely deleterious or that variations in copy number may not always lead to proportional changes in gene expression. Although the correlations between CNV and gene expression are well established in human cancers (Shao et al. 2019; Boen et al. 2021), such associations in Lepidoptera have been reported only for a limited number of genes (Macias-Muñoz et al. 2017; Mulhair et al. 2023). As a result, the extent to which CNVs exert genome-wide deleterious effects via altered gene expression remains uncertain. In contrast, evolutionary conservation through purifying selection by eliminating deleterious nonsynonymous mutations is a universal process across life. Thus, we propose the possibility that changes in copy numbers of the observed iORF-CNVs are more tolerable than changes in amino acid sequences.

The observation that the polymorphic iORF-CNVs originate from genes under weak purifying selection, as indicated by elevated pN/pS, suggests the possibility that genes more tolerant of nonsynonymous point mutations are also more permissive to changes in copy number. Hence, most of the polymorphic iORF-CNVs could be explained by non-adaptive processes. However, it should be noted that we do not exclude the existence of adaptive iORF-CNVs. If directional selection is involved, changes in copy numbers would rapidly be fixed in populations, and we would not see iORF-CNVs as polymorphic within a population. In the case of balancing selection involving alternative protein sequences, an elevation in pN/pS is also possible. It is not possible to estimate the selective coefficients of iORF-CNVs using population genetics analysis to evaluate their fitness effects (Eyre-Walker et al. 2006) because the individually assembled genome sequences were haplotype-collapsed. Estimating the adaptive evolutionary rates involving iORF-CNVs remains a key task and it should certainly be carried out in future studies.

Nonetheless, the nonrandom functional enrichment among iORF-CNVs, particularly in genes related to the chorion and cuticle, suggests that some may have been subject to positive selection. For example, we identified iORF-CNVs in 12 chorion genes, which are involved in eggshell formation in the ovary (Dong et al. 2022). The changes in copy number of chorion genes was also reported in Lepidoptera with the interpretation that it may be linked to adaptation against plant defense responses or hypersensitive response-like necrosis in eggs (Dort et al. 2024). We also observed iORF-CNVs in 12 cuticle genes. It is well known that cuticle thickening is associated with insecticide resistance (Balabanidou et al. 2018), including in *S. litura (*Wang et al. 2025*)*. In *S. frugiperda*, cuticle genes are overexpressed following insecticide treatment (Zhu et al. 2021), further raising the possibility of the adaptive role of these iORF-CNVs.

## Conclusion

In summary, this study investigates the evolutionary origins, mechanisms, and functional conservation of iORF-CNVs in *S. frugiperda* using high-quality, individually assembled genomes. We observed that these CNVs can arise through retrotransposition with *cis-* and *trans-*effects of LINEs. A majority of iORF-CNVs (68.0% to 77.28%) exhibited at least one molecular signature of retrotransposition, indicating that retrotransposition is a primary mechanism for the formation of iORF-CNVs. Other mechanisms may also contribute to the generation of iORF-CNVs; therefore, future studies should investigate the comprehensive mechanisms underlying these variations. We also showed that iORF-CNVs were primarily derived from genes under weak purifying selection while being selectively constrained. Hence, the evolution of iORF-CNVs appears to be different from other CNVs, most of which have lost their function during duplication due to the disruption of the Open Reading Frames. In other words, the primary evolutionary force acting on iORF-CNVs appears to be purifying selection that conserves protein-coding sequences. In contrast, other CNV genes may be more frequently shaped by positive selection favoring the fixation of loss-of-function mutations such as nonsense or frameshift changes, leading to pseudogenization, unless the duplicated copies are eliminated from the population by purifying selection against the CNVs themselves. These non-homogeneous patterns of natural selection highlight the plastic nature of the genome, which is shaped and reshaped by a complex interplay of evolutionary forces, including variation in gene number and function, TE activity, genome structure, and demographic parameters influencing the efficacy of selection.

## Materials and methods

### Establishing bioinformatics pipelines to generate individually assembled genomes

To ensure a sufficient sample size for population-level analysis, we developed bioinformatics pipelines capable of generating high-quality individual genome assemblies from one-sixth of a PacBio HiFi flow cell, providing approximately 10X coverage for the 400 Mb genome. High-molecular-weight genomic DNA was extracted from one half of a single pupa reared from a laboratory colony initiated with corn strain *S. frugiperda* specimens collected in Guadeloupe in 2000 (Gouin et al. 2017) using the QIAGEN DNA Midi Kit according to the manufacturer's protocol. Libraries for PacBio HiFi sequencing were generated with a 15 kb insert size using SMRTbell Express template prep, and sequencing was conducted on the PacBio Sequel II platform.

Subsequently, one-sixth of the total throughput from a single flow cell was allocated for genome assembly using wtdbg2 (Ruan and Li 2020), raven v1.4.0 (Vaser and Šikić 2021), and hifiasm v0.16.1 (Cheng et al. 2021). To polish the assembly, HiFi reads were mapped against the assemblies using minimap2 v2.5 (Li 2018), and racon v1.4.3 (Vaser et al. 2017) was used to improve the assembly with three rounds of iterations.

The quality of the assemblies produced by each assembler was assessed using BUSCO v5.2.2 (Simão et al. 2015) with lepidoptera_odb10 to identify the best assembler and to determine the effectiveness of the polishing iterations. Based on these results, we established a bioinformatics pipeline using the parameters that yielded the highest BUSCO scores to generate high-quality individual genome assemblies from the natural insect population.

### Sample collection and individual genome assembly

Larval sampling was performed by handpicking from an experimental maize field at the University of Florida Institute of Food and Agricultural Sciences Everglades Research and Education Center in Belle Glade, Florida (USA), between October and November 2021. Only one larva was collected per maize plant to minimize kinship among samples. The larvae were shipped alive to the DGIMI laboratory in France and raised to the pupal stage.

The extraction of high-molecular-weight genomic DNA and PacBio HiFi sequencing were performed as described above. A total of 36 samples were sequenced using six flow cells, yielding an average of one-sixth of a flow cell of data per sample, consistent with the procedure established during our pipeline optimization. The *de novo* genome assembly for each sample was conducted following the bioinformatics pipeline established in the previous step, by performing the initial assembly with wtdbg2 and subsequently applying three consecutive rounds of polishing with racon. The quality of the resulting assemblies was subsequently assessed using BUSCO.

To identify potential artificial duplications, self-alignment was performed for each of the 36 assemblies using minimap2 v2-2.28 (Li 2018) with the “-x asm20”' preset. To ensure the detection of large-scale duplications, the bandwidth was set to 100,000 bp using the “−r” option. From the alignment results, sequence pairs longer than 100 kb with a sequence identity exceeding 98% were identified and examined, excluding exact self-matches.

To assess genomic collinearity between the 36 genome assemblies and the ver7 reference genome (https://bipaa.genouest.org/sp/spodoptera_frugiperda/download/genome/spodoptera_frugiperda_corn/v7.0/, also available at https://doi.org/10.5281/zenodo.20487731) (Fiteni et al. 2022), each assembly was mapped against the reference genome using minimap2 with the “-x asm20' preset. Synteny dot plots were then generated using Paf2dotplot v1.0.1 (Jiang 2026), with filtering parameters set to exclude alignment lengths and query sequences shorter than 10 kb. Finally, GraphicsMagick v1.3.38 (GraphicsMagick Group) was used to organize the resulting plots into one layout.

Read depth was calculated in 100 kb windows from the bam files using mosdepth v0.3.11(Pedersen and Quinlan 2018). Only reads with a mapping quality of at least 20 (-Q 20) were included in the analysis. The relative depth of Z chromosomes compared to autosomes was then used to identify the sex of each sample.

### Profiling iORF-CNVs

One protein sequence was selected per gene from the *S. frugiperda* RefSeq annotation (NCBI Genome ID: GCF_023101765.2). These sequences were mapped to each genome assembly using Miniprot v0.13-r248 (Li 2023). Mappings with <95% identity or coverage were discarded, and resulting GFF files were used to extract protein and coding sequences with gffread v0.9.9 (Pertea and Pertea 2020). Any mapping with internal stop codons was also removed. Potential iORF-CNV genes were identified as those with multiple mappings from a single RefSeq protein sequence. This method is robust against the inclusion of pseudogenes or fragmented copies as candidate iORF-CNV genes, as it allows for the direct verification of open reading frame integrity in each genome assembly.

Protein sequences of BUSCO genes (arthropoda_odb10) were mapped to each assembly using Miniprot v0.13-r248 (Li 2023), and mapping counts were recorded per genome. RefSeq protein sequences were also mapped to the ver7 reference genome using the same software. A TE master library was created from the ver7 reference assembly using RepeatModeler v2.0.4 (Flynn et al. 2020). TE copies were located with the resulting library in the ver7 reference assembly using RepeatMasker v4.1.5 (Smit et al. 2013).

HiFi reads were mapped to the ver7 reference genome using minimap2 v2.19 (Li 2018), and read depth was calculated from the resulting bam files using samtools v1.17 (Li et al. 2009). The ver7 reference assembly was generated from a native *S. frugiperda* population sample in Guadeloupe; therefore, the low phylogenetic distance to the field-collected samples is expected to reduce mapping artifacts and subsequently improve the accuracy of variant calling (Lefouili and Nam 2022). Gene Ontology analysis was performed using Blast2GO v 4.1.9 (Conesa and Götz 2008).

To identify the genomic positions of the iORF-CNV insertions, we first extracted a pair of 100 kb flanking sequences for each iORF-CNV gene from each of the 36 individual assemblies. These sequences were mapped to the reference genome using minimap2 v2.19 with the -x asm20 option. An insertion site was validated only if both flanking sequences were successfully mapped to a genomic region spanning a consecutive length of more than 80 kb on the reference genome. We considered an iORF-CNV as being inserted into a non-genic region if the distance between the mapped coordinates of the two inner flanking ends was <100 bp. Shared insertional sites among multiple individuals were then determined by identifying overlapping mapping positions of these flanking regions using Bedtools v2.30.0 (Quinlan and Hall 2010).

### Testing retrotransposition

Inter- or intrachromosomal duplications were inferred based on the chromosomal location of mappings. Chromosome assignments for the contigs of the individually assembled genomes were inferred using the algorithm scaffold of ragtag v2.1 (Alonge et al. 2022), using the ver7 reference genome as a reference. *Trans-*effects of LINEs were inferred from iORF-CNVs flanked by a poly-A sequence (≥20 bp) and target site duplications (≥5 bp). *Cis*-effects were inferred from LINEs located within 100 bp of iORF-CNV genes. TEs in each genome assembly were identified using RepeatMasker v4.1.5 (Smit et al. 2013) with the above-mentioned TE library.

### Testing natural selection

Codon-based alignments of iORF-CNV coding sequences were generated using prank v170427 (Löytynoja 2014), and Gblocks v0.91 (Castresana 2000) was used to remove poorly aligned positions. Divergence at first, second, and third codon positions was calculated with a custom Perl script (see “Data availability”).

From the above-mentioned bam files, haplotype calling was performed, and gVCFs were merged using GATK v4.2.6.1 (McKenna et al. 2010). Variant calling was done using the GenotypeGVCFs function in the same software, and low-quality SNVs were filtered using default parameters. These SNVs were annotated, and synonymous, nonsense, and missense mutations were identified using SnpEff v5.2a (Cingolani et al. 2012).

As in *S. frugiperda*, we identified only one protein sequence for each RefSeq gene in *S. litura* (GCF_002706865.2) and *S. exigua* (GCA_902829305.4), and orthogroups were identified using OrthoFinder v2.5.5 (Emms and Kelly 2019). Illumina resequencing data for *S. exigua* were downloaded from NCBI SRA (accession number: ERR4094912-ERR4094927). Adaptor sequences and low-quality sequences were removed from the reads using AdapterRemoval v3.0.0 (Schubert et al. 2016). Reads were mapped against the reference genome of *S. exigua* (NCBI Genome Accession: GCA_902829305.4) using bowtie v2.5.1 (Langmead and Salzberg 2012), and potential PCR or optical duplicates were removed using picard v2.9.4 (Broad Institute). Haplotype calling was performed using GATK v4.2.0.0 (McKenna et al. 2010), followed by merging the resulting gvcf files into one. Then, variants were called using the GenotypeGVCFs function. Only SNVs were selected from the resulting vcf file, and low-quality SNVs were discarded using the default option. SNVs with the proportion of the called genotype lower than 0.8 were also discarded using vcftools v0.1.16 (Danecek et al. 2011). Synonymous and missense mutations were identified from these SNVs using SnpEff v5.2a (Cingolani et al. 2012), and pN/pS ratio was calculated from the numbers of the synonymous and missense mutations.

### Regulatory sequences of iORF-CNVs

Pairs of the 300 bp upstream regions of iORF-CNVs from a single RefSeq gene were aligned using prank v170427 (Löytynoja 2014). Nucleotide *P*-distances were calculated using a custom Perl script (please see the script availability statement). The lowest distance was recorded for each iORF-CNV copy, and the histogram of these distances was generated using R v4.1.2 (https://www.r-project.org/).

Transcriptome data from *S. frugiperda* samples collected in Florida (Orsucci et al. 2022) were downloaded from the European Nucleotide Archive (Project ID: PRJEB25159). Adapters and low-quality reads were removed using AdapterRemoval v2.1.7 (Schubert et al. 2016). Cleaned reads were mapped to the ver7 reference genome using bowtie2 v2.4.4 (Langmead and Salzberg 2012), and read depth was calculated with samtools v1.17 (Li et al. 2009). TATA-box motifs were identified using Perl regular expressions (∼/TATA[A|T]A[A|T]/).

### Synteny analysis

Conserved synteny between *S. frugiperda*, *S. litura*, and *S. exigua* was inferred using DAGchainer r02-06-2008 (Haas et al. 2004) with the protein sequences used to identify orthogroups. More specifically, these protein sequences were “blastp”ed using BLAST 2.12.0+ (Camacho et al. 2009), and the position of corresponding genes was recorded. Then, the run_DAG_chainer.pl script within the DAGchainer package was executed to identify the position of orthologous gene pairs between the two species among the three species. The BUSCO lepidoptera_odb10 proteins were “blastp”ed against the used protein sequences of *S. frugiperda*, and the position of the BUSCO genes was identified from the DAGchainer result. Then, the orthologous chromosomes among the three species were identified. The positions of orthologous BUSCO genes were visualized using the circlize v0.4.17 (Gu et al. 2014). We identified the position of intronless genes to list the deviation from the conserved synteny.

## Supplementary Material

msag124_Supplementary_Data

## Data Availability

Raw HiFi resequencing data and individually assembled genomes generated in this study are available at NCBI (Project Number: PRJNA1269917). Individually assembled genomes and gene annotations are also available at the BioInformatics Platform for Agroecosystem Arthropods (https://bipaa.genouest.org/sp/spodoptera_frugiperda_pub/download/genome/corn/v7.0/). All computer programming scripts used in this study are available at GitHub (https://github.com/kiwoong-nam/sfrugi_WG).
