# Supplementary material for: Retrotransposed gene copies persist under relaxed selection in wild Spodoptera frugiperda
Source: Mol Biol Evol. 2026 May 27;43(6):msag124. doi: 10.1093/molbev/msag124 (PMC13251582; doi:10.1093/molbev/msag124)
Supplement: msag124_Supplementary_Data [file msag124_supplementary_data.zip › rerevision.sfrugi_WG_Supple.docx]

**Supporting Information for**

**Retrotransposed gene copies persist under relaxed selection in wild *Spodoptera frugiperda***

Kiwoong Nam^1*^, Sylvie Gimenez^1^, Hyerin An^1^, Karine Durand^1^, Melanie Gasser^1^, Sudeeptha Yainna^1^, Fabrice Legeai^2^, Julien Beuzelin^3^, David G. Heckel^4^, Sabine Hänniger^4,5^, Emmanuelle d'Alençon^1^

^1^DGIMI, INRAE, Univ Montpellier, Montpellier, France

^2^INRAE, UMR-IGEPP, BioInformatics Platform for Agroecosystems Arthropods, Campus Beaulieu, Rennes, 35042, France

^3^Everglades Research & Education Center, University of Florida Institute of Food and Agricultural Sciences, Belle Glade, FL 33430, USA

^4^Entomology Department, Max Planck Institute for Chemical Ecology, Jena, Germany

^5^Institute for Biodiversity, Ecology and Evolution, Friedrich Schiller University Jena, Jena, Germany

*corresponding author: [ki-woong.nam@inrae.fr](mailto:ki-woong.nam@inrae.fr)

**This Supporting Information includes.**

Figure S1 - Figure S13

Table S1, S4


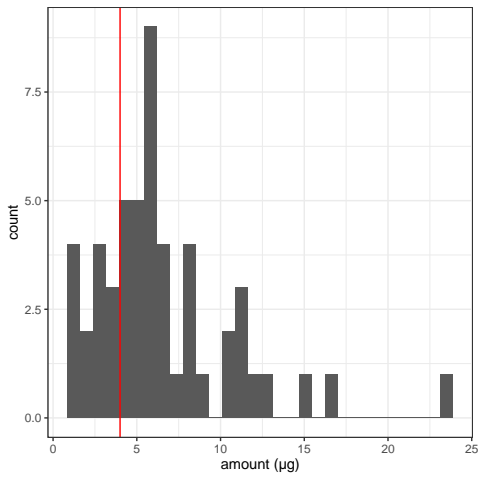
Figure S1. Distribution of the amount of gDNA extracted from each single individual across 52 samples. The red vertical bar indicates 4 µg, which was used as the threshold to proceed with PacBio HiFi sequencing.

Figure S2
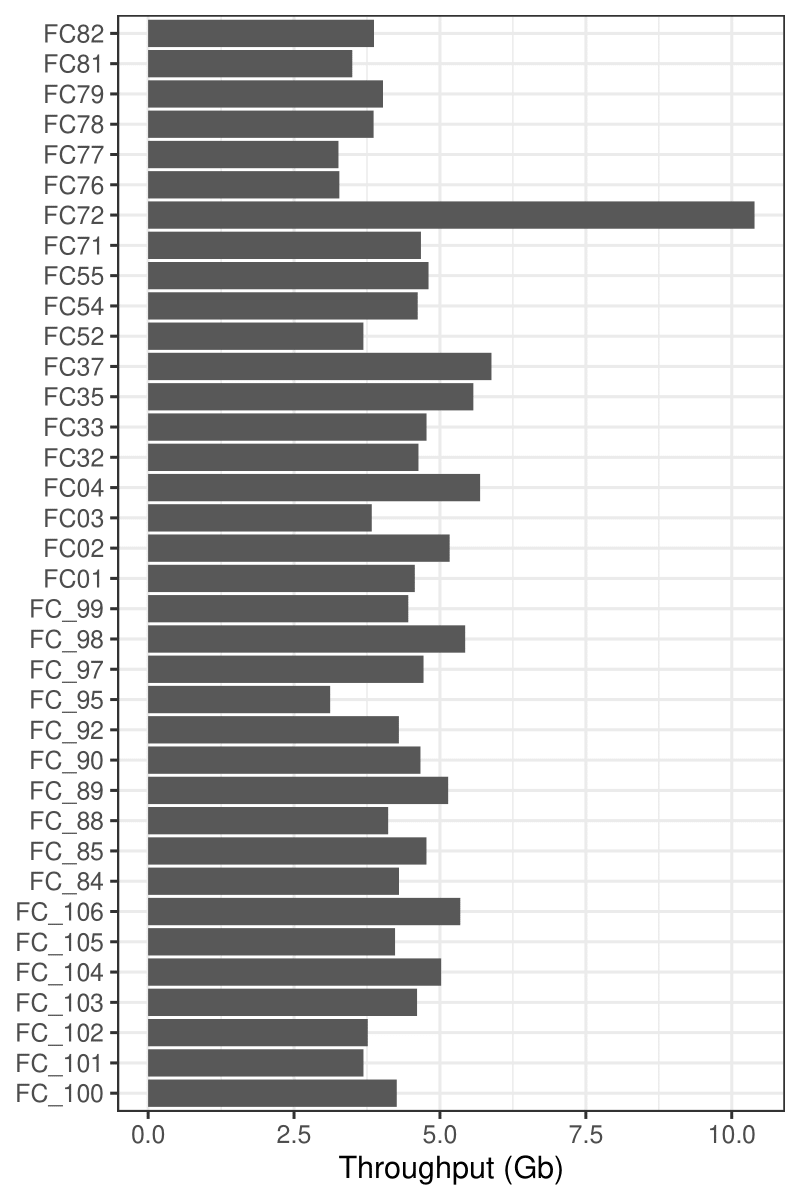
. The throughput of sequencing for each sample.


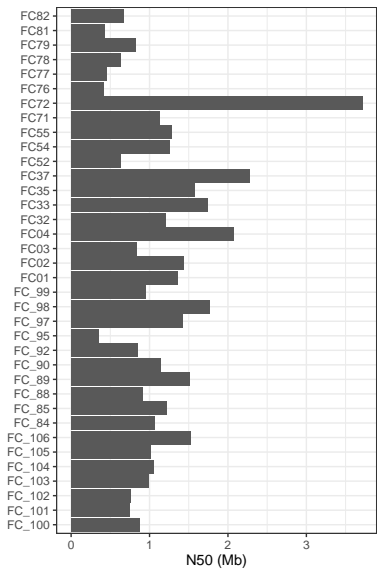
Figure S3. N50 of each individually assembled genomes


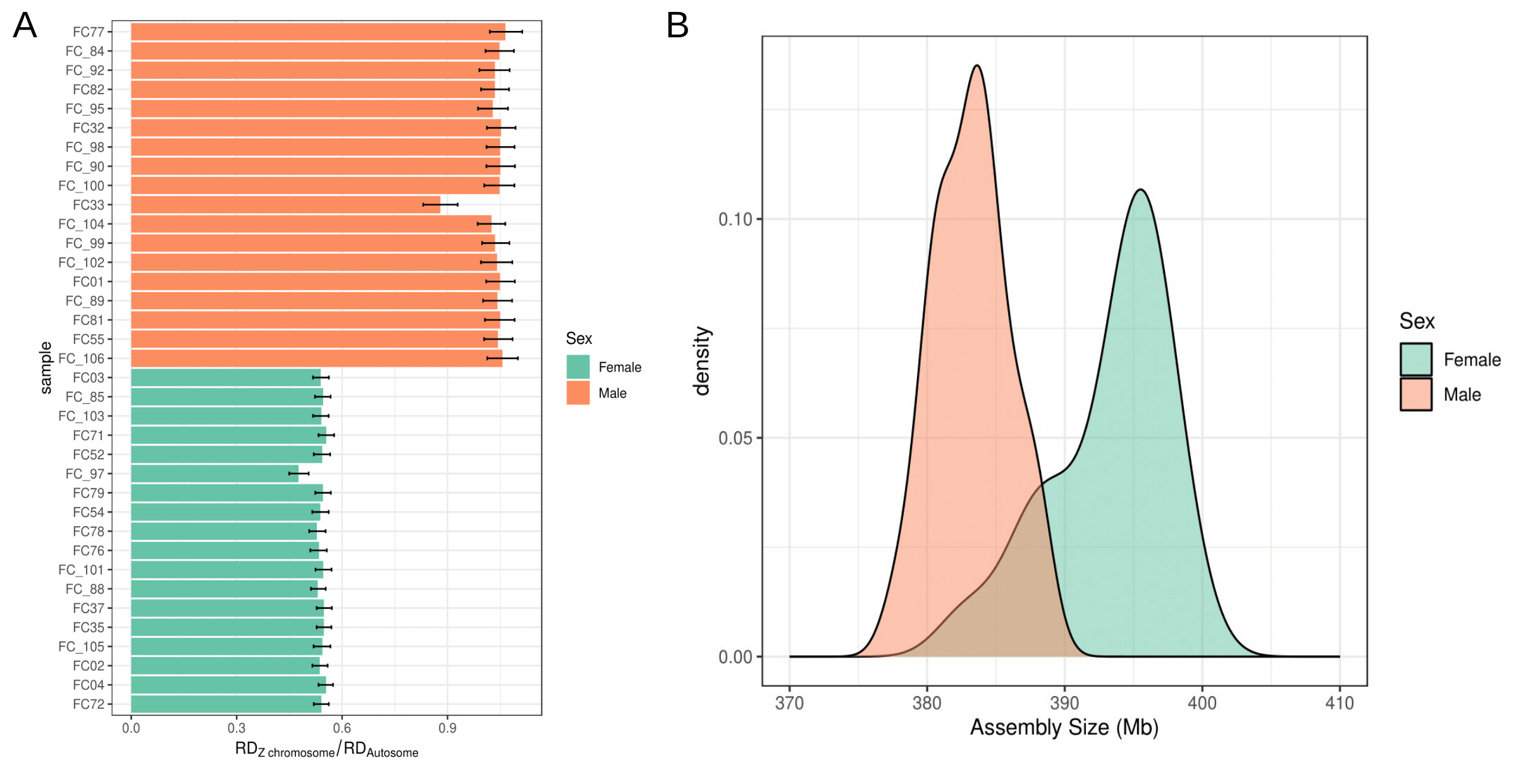
Figure S4. Assembly size variation by sex. A. The relative read depth of HiFi reads of the Z chromosomes to the autosomes in the reference genome assembly. If this relative read depth is close to 1 or 0.5, corresponding samples were considered to be males or females, respectively. B. The assembly sizes according to the determined sex. Females had larger assembly sizes than males.


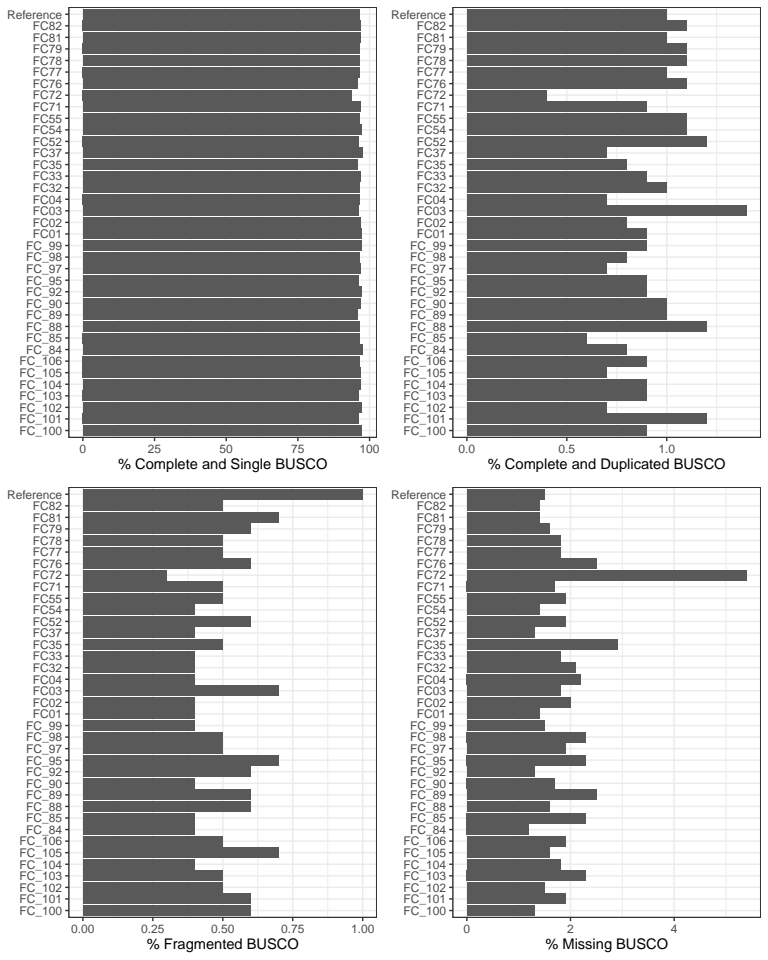
Figure S5. Proportions of Complete and Single-copy, Complete and Duplicated, Fragmented, and Missing BUSCO genes across 36 assemblies from field-collected samples and the reference genome assembly.


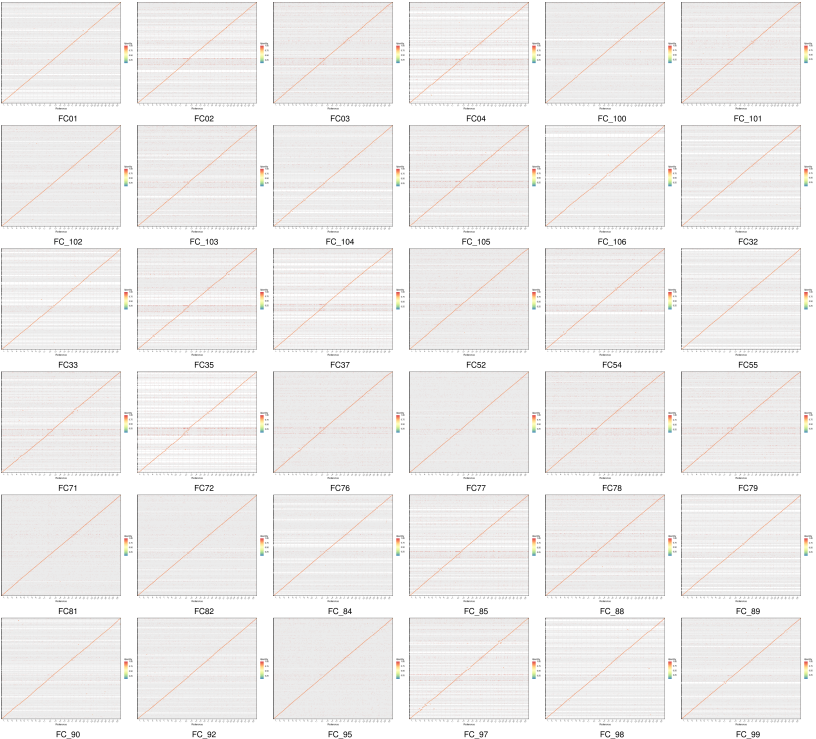


Figure S6. The dotted plot showing the collinearity between the reference genome assembly and each assembly from 36 samples. The color of the dots shows the sequence identify.

Figure S7
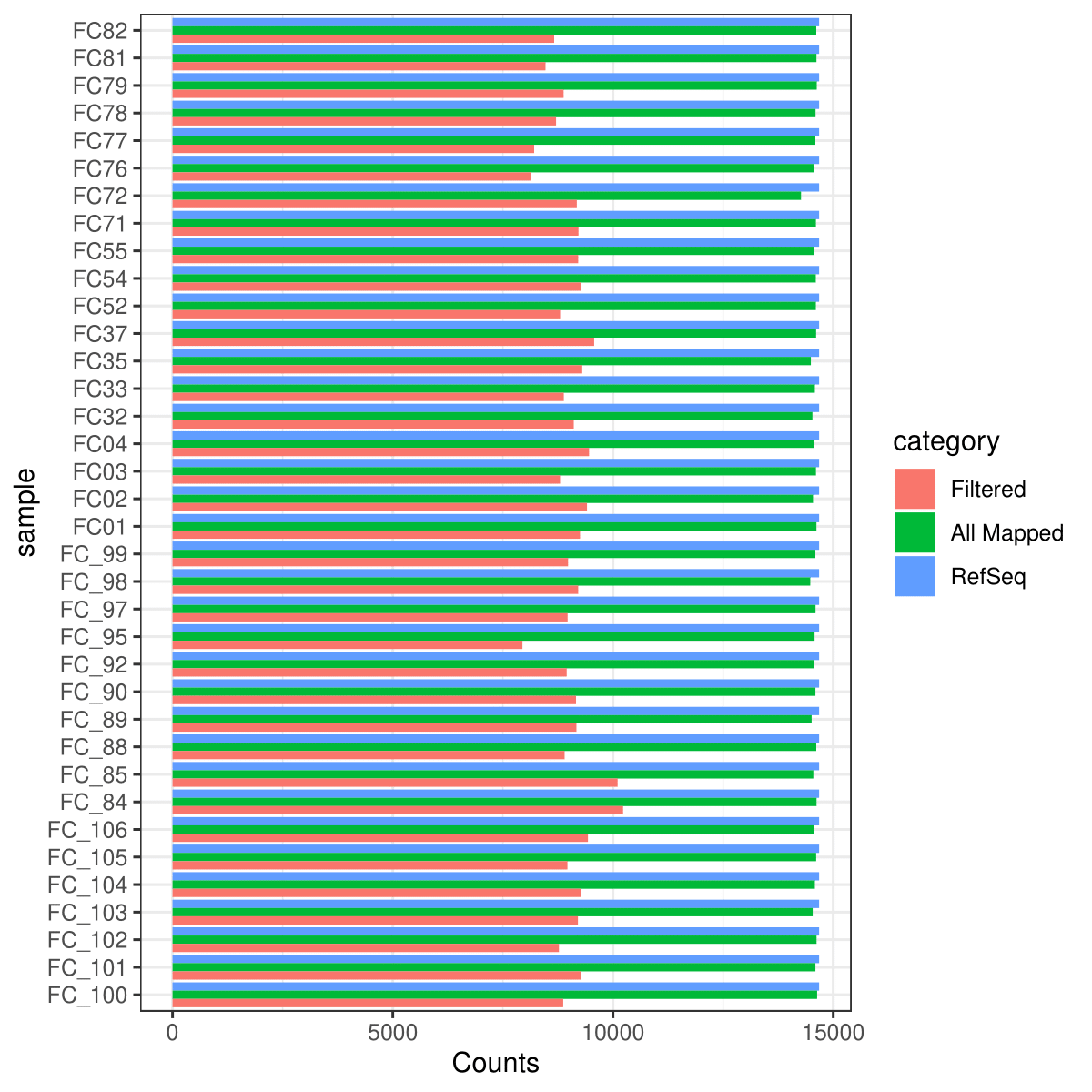
. Total number of RefSeq genes, number of mapped RefSeq genes for each sample, and number of filtered RefSeq genes for each sample.

Figure S8. Histogram showing the number of samples with a second CNV allele, indicating the presence of duplicated genes within a genome
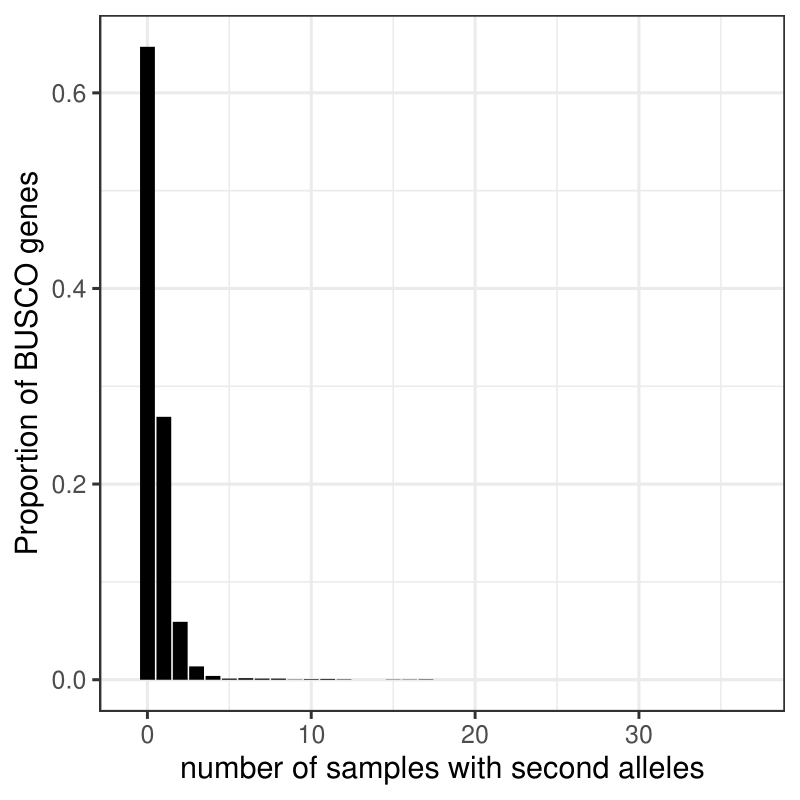
, for each BUSCO gene.


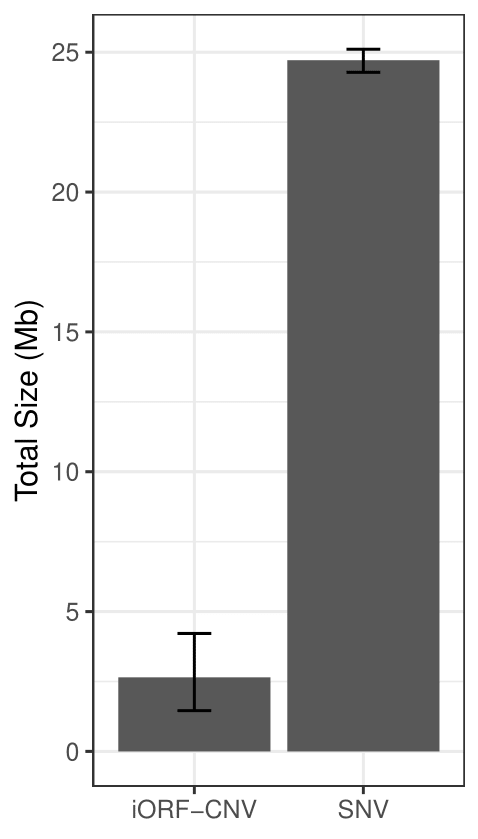


Figure S9. The total sizes of RefSeq sequences showing iORF-CNVs or Single Nucleotide Variations (SNV).


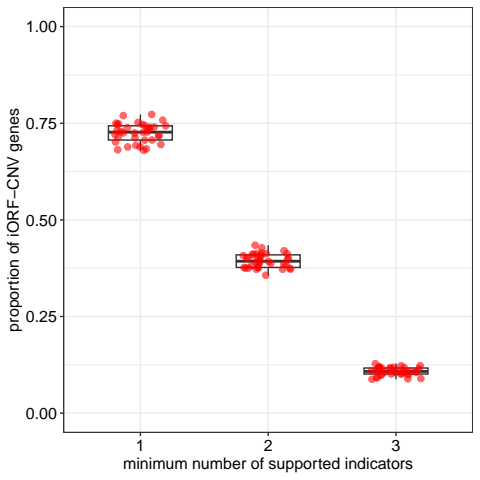


Figure S10. Proportion of iORF-CNV genes with the minimum number of supporting indicators for retrotransposition including intronless structure, interchromosomal duplication, and LINE-mediated insertion footprints.


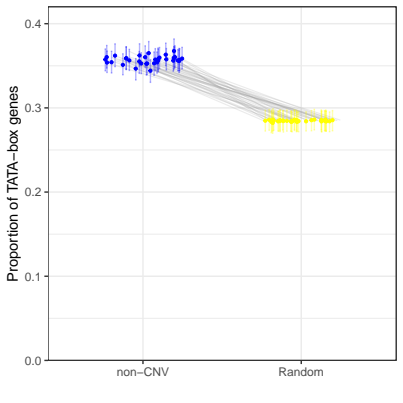
Figure S11. The proportions of TATA-box motifs of (blue) non-iORF-CNV genes and (yellow) 1,000 randomly chosen sequences for each genome assembly.


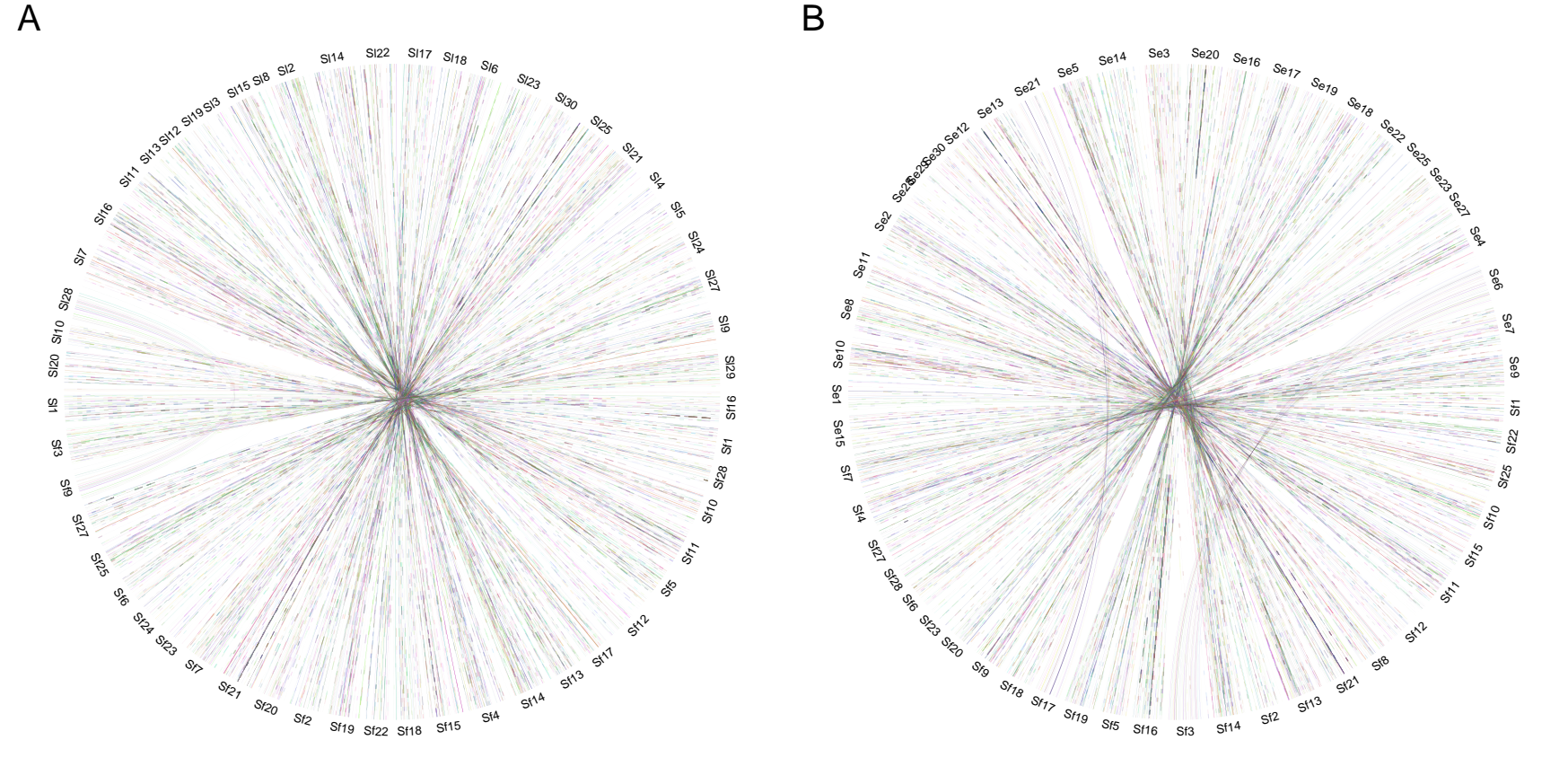
Figure S12. Circular visualization of genomic synteny between *Spodoptera frugiperda* and (A) *S. litura* or (B) *S. exigua*. The outer labels indicate the chromosomes of each species. Coloured ribbons represent orthologous blocks identified via DAGChainer, with ribbons connecting conserved genomic positions between the two genomes.


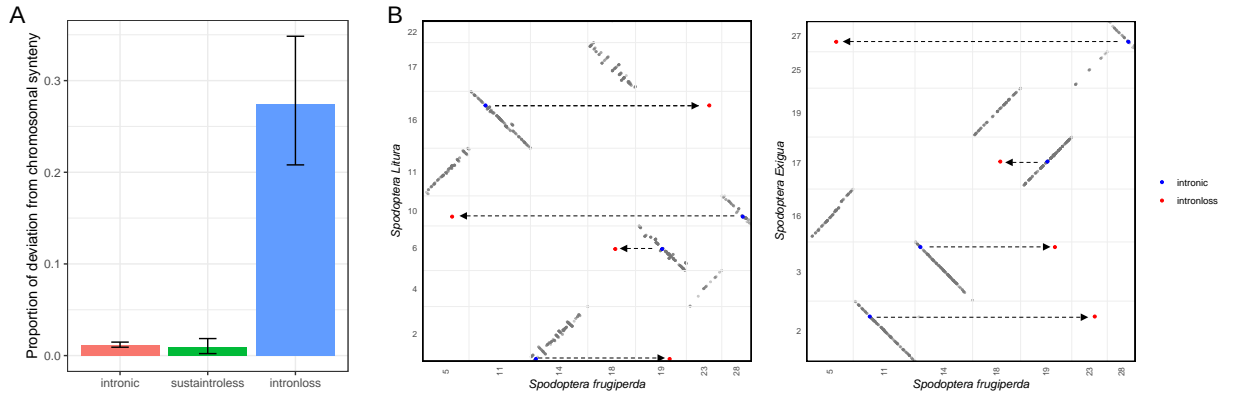


Figure S13. Evolutionary analysis of gene duplication involving intron loss. A. Proportions of genes showing synteny deviation across different gene categories. The bar plot compares *(i)* intronic genes, *(ii)* sustaintronloss (those maintaining an intronless state from the common ancestor of *S. frugiperda*, *S. litura*, and *S. exigua*), and *(iii)* intronlost genes (those that lost introns after the divergence of *S. frugiperda* and *S. litura*) in the lineage of *S. frugiperda*. Deviations were calculated based on the syntenic relationships of orthologous chromosomes among the three *Spodoptera* species. The error bars represent 95% bootstrapping confidence intervals calculated by resampling the orthogroups. B. Examples showing that gene duplication involving intron loss is associated with a deviation from ancestral synteny. Blue and red dots represent intronic genes (ancestral status) and intronless genes (derived status), respectively. Gray dots represent BUSCO genes used to establish the syntenic background. Dashed arrows indicate the relationship between the original intronic gene and the duplicated intron-losing copy within the same orthogroup.

Table S1. BUSCO scores of genome assemblies generated from one-sixth of the throughput of a single PacBio HiFi flow cell using various genome assemblers.

| Assembler | Complete | Single | Duplicated | Fragmented | Missing | Total |
| --- | --- | --- | --- | --- | --- | --- |
| wtdbg2 | 5,170 | 5,148 | 22 | 27 | 89 | 5,286 |
| hifiasm | 5,152 | 4,793 | 359 | 26 | 108 | 5,286 |
| raven | 4,913 | 4,867 | 46 | 62 | 311 | 5,286 |

Table S4.The list of overrepresented GO terms in iORF-CNV genes

| GO terms | GO Category | GO Name | FDR-corrected  p value | Odd  ratio |
| --- | --- | --- | --- | --- |
| GO:0007304 | BIOLOGICAL_PROCESS | chorion-containing eggshell formation | 3.32 × 10^-14^ | 322.49 |
| GO:0071897 | BIOLOGICAL_PROCESS | DNA biosynthetic process | 1.23 × 10^-74^ | 256.10 |
| GO:0015074 | BIOLOGICAL_PROCESS | DNA integration | 2.45 × 10^-47^ | 376.84 |
| GO:0006334 | BIOLOGICAL_PROCESS | nucleosome assembly | 2.21 × 10^-2^ | 38.63 |
| GO:2000767 | BIOLOGICAL_PROCESS | positive regulation of cytoplasmic translation | 7.07 × 10^-4^ | 103.50 |
| GO:0032197 | BIOLOGICAL_PROCESS | transposition, RNA-mediated | 3.13 × 10^-3^ | Inf |
| GO:0042600 | CELLULAR_COMPONENT | chorion | 3.32 × 10^-14^ | 322.49 |
| GO:0042575 | CELLULAR_COMPONENT | DNA polymerase complex | 1.11× 10^-32^ | 121.17 |
| GO:0000786 | CELLULAR_COMPONENT | nucleosome | 2.12× 10^-6^ | 156.70 |
| GO:0004190 | MOLECULAR_FUNCTION | aspartic-type endopeptidase activity | 6.62× 10^-3^ | 25.86 |
| GO:0046983 | MOLECULAR_FUNCTION | protein dimerization activity | 1.12× 10^-5^ | 6.10 |
| GO:0003727 | MOLECULAR_FUNCTION | single-stranded RNA binding | 1.89× 10^-4^ | 16.67 |
| GO:0005213 | MOLECULAR_FUNCTION | structural constituent of chorion | 3.32× 10^-14^ | 322.49 |
| GO:0030527 | MOLECULAR_FUNCTION | structural constituent of chromatin | 2.12× 10^-6^ | 156.70 |
| GO:0042302 | MOLECULAR_FUNCTION | structural constituent of cuticle | 3.02× 10^-3^ | 4.22 |
| GO:0045182 | MOLECULAR_FUNCTION | translation regulator activity | 3.26× 10^-2^ | 12.92 |
| GO:0008270 | MOLECULAR_FUNCTION | zinc ion binding | 7.44× 10^-4^ | 3.05 |
